# Glands of Moll: history, current knowledge and their role in ocular surface homeostasis and disease

**DOI:** 10.1016/j.preteyeres.2025.101362

**Published:** 2025-05-05

**Authors:** Michael Stopfer, Ingrid Zahn, Katharina Jüngert, Gerhard Aumüller, Frans L. Moll, Martin Schicht, Helen P. Makarenkova, Cintia S. de Paiva, Friedrich P. Paulsen

**Affiliations:** a Institute of Functional and Clinical Anatomy, Friedrich-Alexander-University Erlangen-Nürnberg, Universitätsstr. 19, Erlangen, Germany; b Philipps-University Marburg, Am Möhrengarten 1, 35117, Münchhausen, Germany; c Department of Vascular Surgery, University Medical Center Utrecht, Heidelberglaan 100, 3584, CX, Utrecht, the Netherlands; d Department of Molecular Medicine, The Scripps Research Institute, 10550 N Torrey Pines Rd, La Jolla, CA, 92037, United States; e Ocular Surface Center, Department of Ophthalmology, Baylor College of Medicine, One Baylor Plaza, Houston, TX, 77030, United States

**Keywords:** Jacob Anthonie Moll, Moll glands, Ciliary glands, Glandulae ciliares conjunctivales, Sweat gland, Apocrine gland, Ocular surface, Lid margin, Tear film

## Abstract

Over the last 20 years, research into the Meibomian glands of the eyelids has increased exponentially and is now widely recognized as a field of research. It is all the more astonishing that knowledge about another type of gland in the eyelids, the Moll glands or ciliary glands, has almost stagnated and there has been little to almost no progress, even though this type of gland as a whole takes up a relatively large volume in the upper and lower eyelids. There is not much information about the namesake Moll or the function of the glands although these are listed in nearly every textbook of anatomy, histology and ophthalmology. For this reason, we set out to compile the existing knowledge about the Moll glands of the eyelids in order to create a basis for follow-up studies and to stimulate research into this type of gland. In our literature research, we went back to the middle of the 19th century and made contact with a descendant of the Moll family and illustrate their relevance for the present. The structure of the secretory part of the Moll glands is very well described, a number of secretory products are known, but the current state of research allows only very rough speculations about their function. The overview provides numerous interesting insights, which, however, raise more questions than they provide answers.

## 1. Introduction

The lacrimal functional unit (LFU) is a highly complex and dynamic system that ensures the maintenance, regulation, and stability of the tear film, which is essential for ocular surface health and visual function. It comprises multiple interconnected components, including the lacrimal glands, the ocular surface (cornea and conjunctiva), the eyelids and their associated glands (such as Meibomian and accessory lacrimal glands), as well as the complex network of sensory, autonomic, and motor nerves that coordinate their function (Stern et al., 2004). The LFU continuously responds to environmental stimuli, hormonal fluctuations, and neural inputs, integrating sensory feedback with efferent signaling to modulate tear production, composition, and distribution. This dynamic regulation helps maintain ocular surface integrity, prevent desiccation, and support the immune defense of the eye. Dysfunction in any part of the LFU can lead to tear film instability, inflammation, and ocular surface disorders, highlighting its critical role in ocular health (Stern et al., 2004).

Extensive research has been done on the lacrimal glands as well as on the Meibomian glands in the eyelids (the latter have experienced a veritable scientific boom in the last 20 years) (Knop et al., 2011). However, despite this progress, our understanding of the numerous other glands within the eyelids, such as the glands of Zeis, ciliary (Moll), as well as Krause and Wolfring, remains limited (Khan et al., 2022). These lesser-studied glands, though smaller in size, play vital roles in maintaining ocular surface health by contributing to tear film stability and antimicrobial defense. This applies in particular to the ciliary glands or eyelash glands (Glandulae ciliares conjunctivales, Glandulae tarsals ciliares) named after Jacob Anthonie Moll, which are closely associated with the eyelashes. Each eyelash has its own ciliary gland (Moll gland, Glandula tarsalis ciliaris, Glandula ciliaris conjunctivalis), so that the entire eyelid margin is fully occupied by Moll glands. Given the ongoing debate regarding whether Moll glands open into the cilia of the eyelashes or open directly onto the lid margin, whether they represent a distinct type of gland and whether their secretion contributes to the tear film, this review aim is to summarize the existing knowledge on the distribution and function of the Moll glands on the ocular surface in order to provide a research basis for their role in tear film homeostasis. By doing this, our review seeks to establish a foundation for future research into their potential role in tear film homeostasis. The function of the Moll glands is particularly intriguing, as diseases affecting the eyelid margin are highly very common, and the eyelid margin is vital to ocular surface integrity (Yeu et al., 2024). Additionally, there is limited information about Jacob Anthonie Moll on the internet, which is why we set out to find out more about him and his contemporaries who also worked on the ciliary glands.

## 2. Jacob Anthonie Moll

**Fig. 1.**
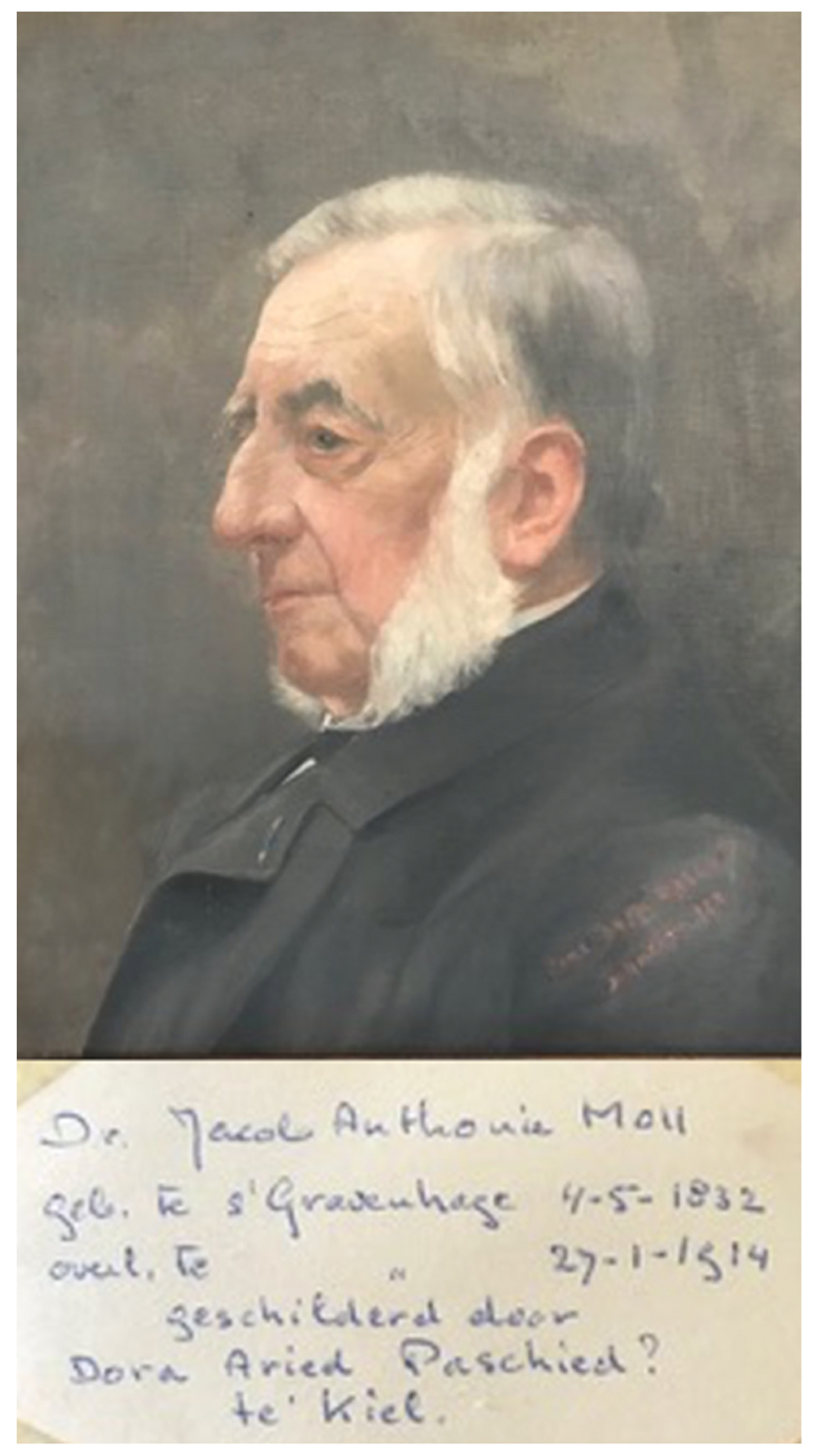
Jacob Anthonie Moll aged around 65, private property of the Moll family (Jaap Moll, second cousin of coauthor Frans Moll). At the bottom reference to the portrait painter: “painted by Dora Aried Paschied ? in Kiel.

**Fig. 2.**
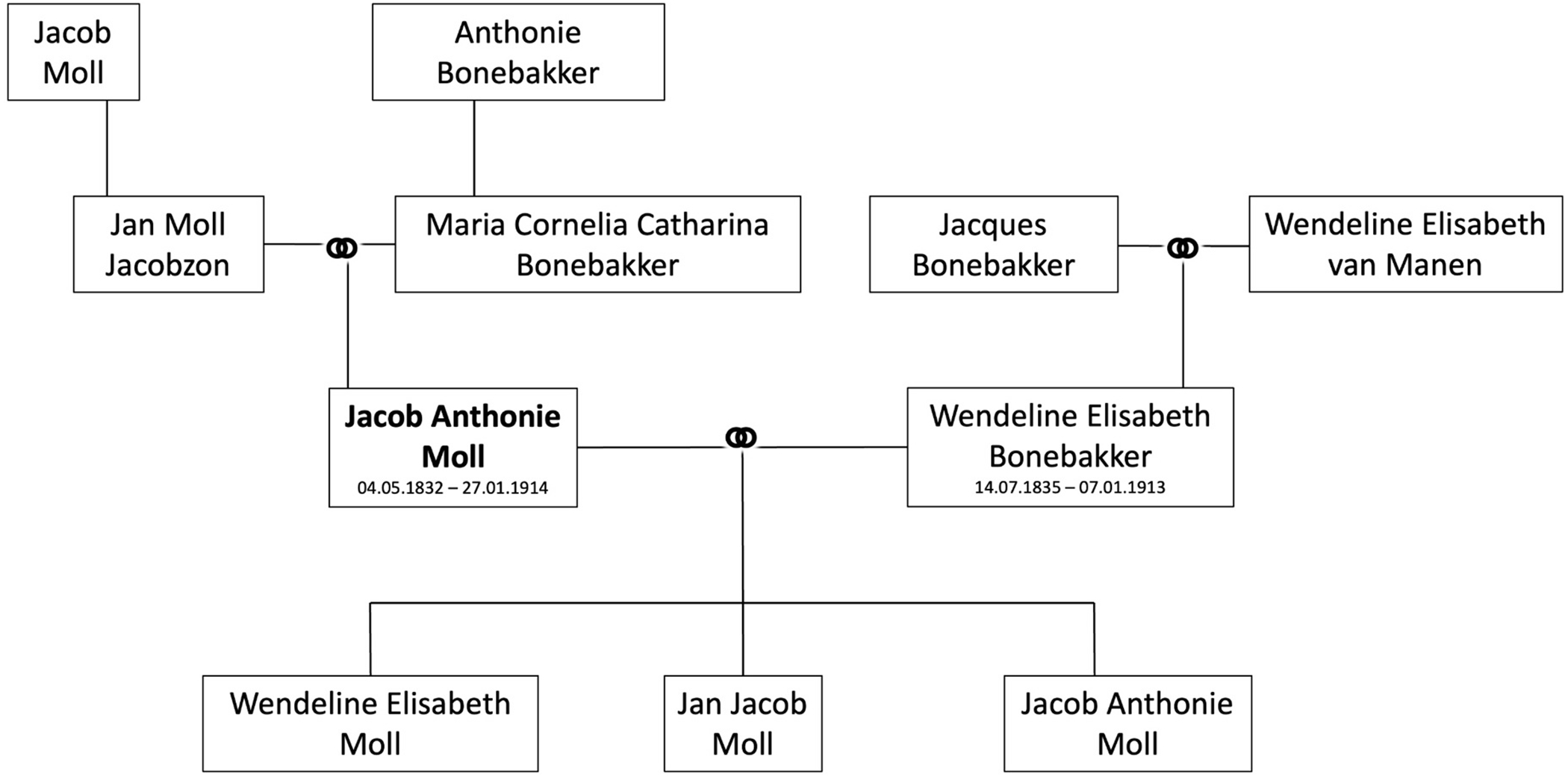
Family tree of Jacob Antonie Moll (shown in bold). [The youngest son of Jacob Anthonie Moll after whom the gands of Moll are named bore the same name as hist farther].

The human Moll glands are named after Jacob Anthonie Moll, a Dutch physician (Fig. 1). He was born on May 4, 1832 in The Hague and was the eldest of eight children of Jan Moll Jacobszon, a preacher (a lay preacher who has undergone special theological training, writes his own sermons, and preaches them freely within the Protestant parishes) in the Low German Reformed congregation (Fig. 2). His mother was Maria Cornelia Catharina Bonebakker (Suppl. Fig. 1). He was therefore part of the Moll-Blokzijl family, known for producing several distinguished scholars and professionals. Jacob Anthonie Moll was named after both his paternal grandfather, Jacob Moll, and his maternal grandfather, Anthonie Bonebakker (Fig. 2). He married Wendelina Elisabeth Bone-bakker (*14.07.1835), daughter of Jacques Bonebakker and Wendeline Elisabeth van Manen (Fig. 2), on May 31, 1860 in Amsterdam (Suppl. Fig. 2). The marriage produced three children: Jan Jacob Moll (*16.8.1861, The Hague), Wendelina Elisabeth Moll (*15.3.1863, The Hague) and Jacob Anthonie Moll (*20.8.1872, The Hague) (Fig. 2) [The latter thus bore the same name as his farther, after whom the glands of Moll are named].

**Fig. 3.**
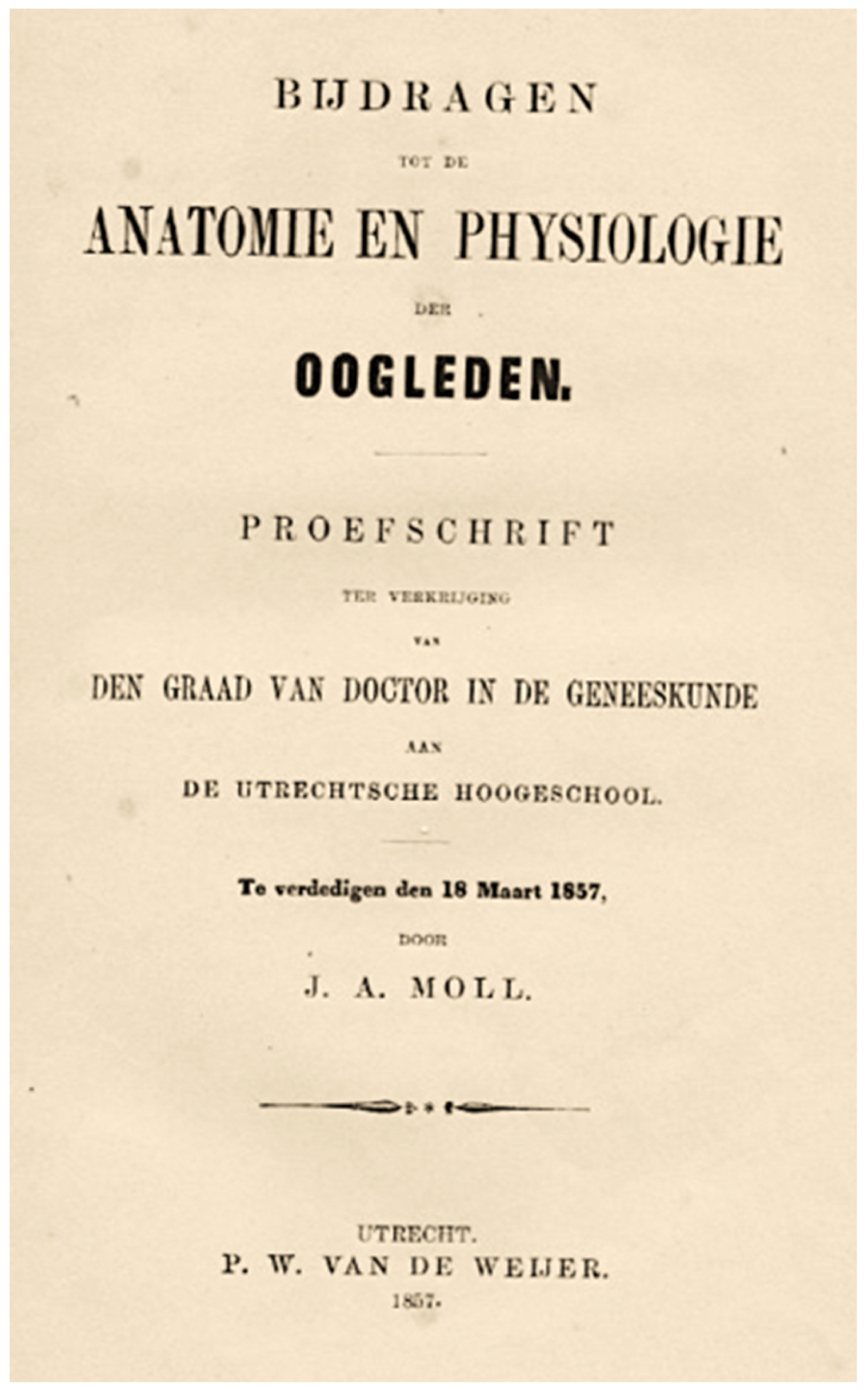
Title page of the dissertation by Jacob Anthonie Moll (1957): Translation: Contributions to the anatomy and physiology of the eyelids - Dissertation for the degree of Doctor of Medicine at the University of Utrecht - defended on March 18, 1857 by J. A. Moll - Utrecht - P.W. van de Weijer 1875.

From 1850, Jacob Anthonie Moll studied medicine at the University of Utrecht/Netherlands. In June 1854, Moll became an assistant to Professor F. C. Donders,^1^ passed the candidate examination in medicine in March 1855, was elected to the Rectorate Senatus Veteranorum and passed the doctoral examination in June 1856. On March 18, 1857, J. A. Moll finally received his doctorate in Utrecht with the title of his work ‘Bijdragen tot de Anatomie en Physiologie der Oogleden’ (Contributions to the anatomy and physiology of the eyelids) (Fig. 3). In this dissertation he describes the general construction of the eyelids, the eyelashes, the tarsus and the Meibomian glands, the conjunctiva and the corpus papillare, the muscles and the movements of the eyelids. He also describes the small glands between the eyelashes, which were later named after him (Moll, 1857).

^1^

In August 1857, Moll was appointed to the Buitengasthuis in Amsterdam, where he obtained a second doctorate in surgery on April 17, 1858 under the mentorship of the surgeon Prof. van Ittersum in Amsterdam. At the age of 26, Moll then opened his own practice in The Hague/Netherlands and worked here until his retirement on December 31, 1901. Jacob Anthonie Moll died at the age of 81 on January 27, 1914 in The Hague (Suppl Fig. 3).

To summarize and bring it up to date, Jacob Anthonie Moll was a doctoral student of Professor F. C. Donders,^1^ who researched the anatomy of the eyelids and published his findings in a journal relevant to the time due to Donders’ reputation. After completing his doctoral thesis, he became a practicing doctor like most of his colleagues at the time.

## 3. Moll’s description of the glandulae ciliares conjunctivales in the context of his time

Although the glandulae ciliares conjunctivales (glandulae tarsals ciliares) are named after Moll, they were described by Albert von Kölliker (1817–1905, professor of anatomy in Würzburg) as early as 1850 as ‘sweat glands of conspicuous appearance, which frequently open into the upper end of the hair follicles of the eyelashes’ (Kölliker, 1850).

In his doctoral thesis (Moll, 1857) and in a subsequent publication (Moll, 1858), Moll repeatedly emphasized that the glands had already been described by A. Kölliker. Moll describes in particular the peculiar shape of the ciliary glands on the eyelid and points out that the glands consist of only a single convoluted canal. In the years that followed, the glands received little attention. Only Henle (1871) points out that the bulbous glands - as he calls them - are slightly larger on the lower eyelid than on the upper eyelid. Sattler (1844–1928), an Austrian-German ophthalmologist and professor of ophthalmology in Vienna, was the first to conduct an in depth study of the ‘modified (Moll’s) sweat glands at the eyelid margin (Sattler, 1877). He acknowledged that both Kölliker and Moll had previously described these glands; however, through the title of the publication he effectively lays the foundation for the later name ‘Moll glands’. In his article, he shows that the glands have almost been forgotten since Moll’s description in 1857 and presents his own intensive investigations into the glands. In particular, he discusses the excretory ducts of the glands and points out that these do not (or only very rarely) open directly at the skin of the eyelid margin, but open into the hair funnel of the eyelashes just above the sebaceous gland orifices (Zeis glands, Glandulae sebaceae) (and thus closer to the free eyelid margin). He also describes the position of the Moll glands very precisely. Their ends reach between the muscle bundles of the M. ciliaris Riolani (Riolan muscle) or the lowest bundles of the tarsal portion of the orbicularis eye muscle and speculates that the glandular tubes must be compressed slightly with each blink. According to Sattler, the glands are surrounded by a dense blood vessel system. He also describes the myoepithelial cells surrounding the glands, referring to them as ‘a simple layer of smooth muscle fibers’ that extend to the excretory duct but never merge with it. In his 1877 study, Sattler further notes the presence of highly refractive, yellow-brown granules in the glandular cells, which can reach diameters of up to 0.001 mm in adults but are absent in children. The multilayered epithelium of the excretory duct is clearly different; the width of the excretory duct clearing is proportionally larger in children than in adults. In newborns, he sees all the conditions described above already fully developed. Compared to other sweat glands, the development of the Moll glands is significantly more advanced. Sattler was thus the first to focus intensively on the modified (Moll glands) sweat glands of the eyelid margin (Sattler, 1877).

Subsequent studies on the Moll glands were conducted by Waldeyer (1874), Königstein (1884), Contino (1907), Ask (1908) Virchow (1922), Peter and Horn (1935) as well as Tabara (1953). However, these investigations did not yield any significant new insights. Comparative anatomical studies were carried out by Tartuferi (1879), Eggeling (1904), Zietzschmann (1904) and Schaffer (1940), but it was not until Ikeda (1953) that the distribution and size of the ciliary glands (Moll glands) in various mammals was specifically investigated (Table 1). In this context it is noteworthy that common laboratory animals such as mice, rats and rabbits do not appear to have Moll glands, whereas they are found in slightly larger species such as cat, dog or pig (Table 1).

**Fig. 4.**
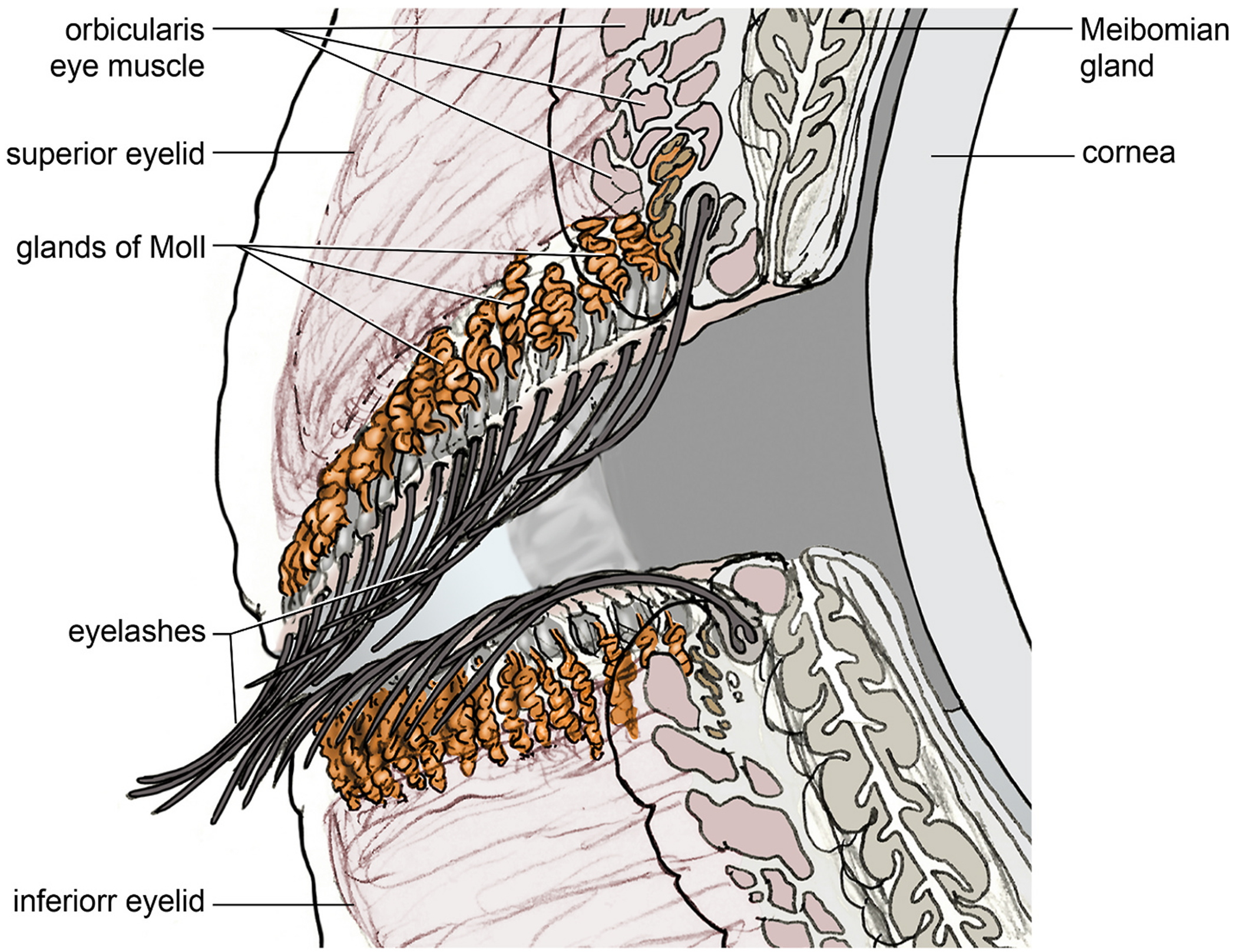
Distribution of the Moll glands along the upper and lower eyelid (drawing by Jörg Pekarsky). The illustration shows that the totality of all Moll glands in the upper and lower eyelids results in an extensive glandular volume, which in its entirety accounts for more volume than the main lacrimal gland.

In addition, Ikeda (1953) was able to show that, with the exception of the pig, the ciliary glands (Moll glands) are the most pronounced in humans and make up a large amount of glandular tissue in their entirety (Fig. 4).

**Fig. 5.**
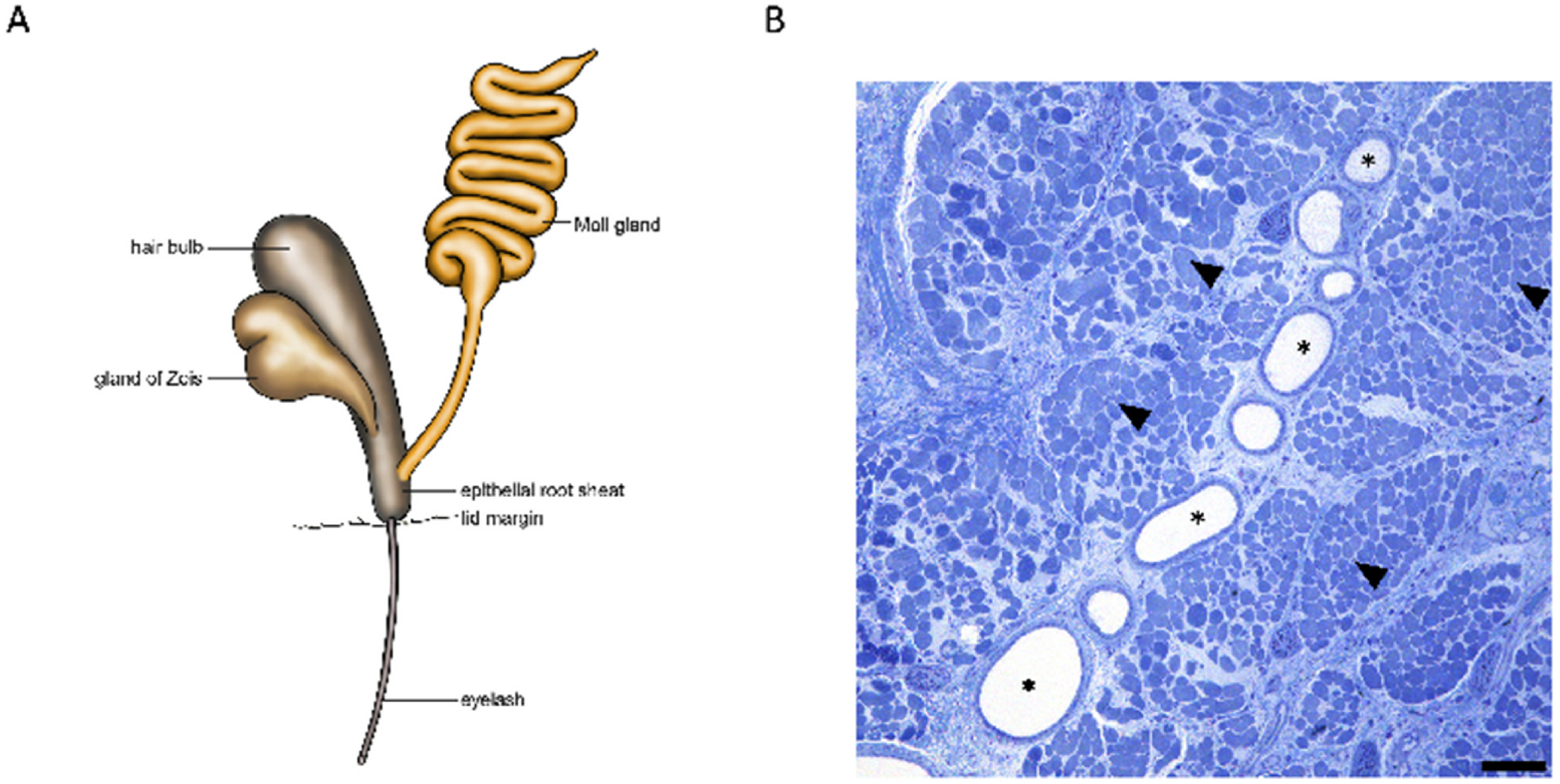
Depiction of a Moll gland (gland of Moll) on the hair funnel (epithelial root sheet). (drawing by Jörg Pekarsky) and histological representation of the secretory part of a Moll gland between the muscle fibers of the Riolan muscle. **A** shows a “*Moll gland unit*”. This consists of a proximal nodular secretory glandular part and a ductal part. The proximal part lies partly between muscle fibers of the Riolan muscle (arrowheads in **B**, distal part of the orbicularis oculi muscle, whose function has not been conclusively clarified). The distal part of the Moll gland runs straight towards the hair bulb of an eyelash (**A**) and joins the epithelial root sheet of the eyelash distally before the junction of a gland of Zeis (= sebaceous gland) but before the lid margin. **B** shows the secretory part (stars) of a Moll gland between the Riolan muscle (arrowheads). Image with Toluidine blues staining, scale bar 100 μm.

Histologically, Moll glands are categorized as apocrine sweat glands (Lüllmann-Rauch and Asan, 2024). An apocrine gland unit consists of a proximal nodular secretory glandular part and a ductal part, the main part of which runs through the dermis to the skin surface. The difference with the Moll glands (Moll gland units) is that the excretory duct opens into the hair funnel of the eyelashes and does not or only very rarely open at the skin surface (Sattler, 1877) (Fig. 5A).

**Fig. 6.**
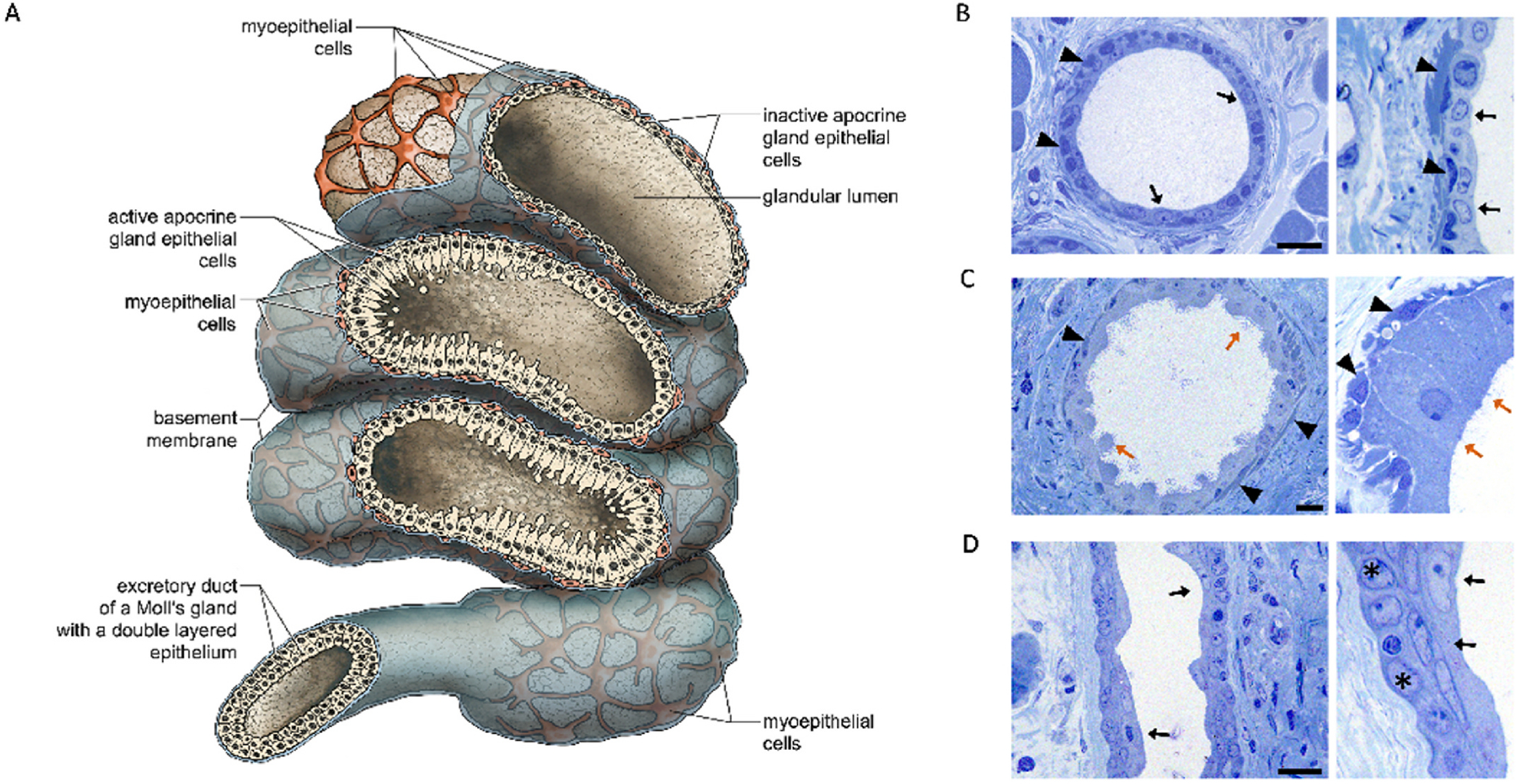
A illustration of an opened Moll gland with secretory part and excretory duct part. The various functional states of the Moll epithelial cells and the wide glandular lumen can be seen in the secretory part. The secretory part is surrounded by myoepithelial cells. (Drawing by Jörg Pekarsky). **B** The Moll gland with a single layer of inactive/flat glandular cells (arrows) and myoepithelial cells (arrowheads). **C** The Moll gland with a single layer of active secretory cells (red arrows), and myoepithelial cells (black arrowheads). Active apocrine gland cells with high apical cells (brown arrow) and glandular vesicles in the lumen. **D** The excretory ducts consist of two cell layers made up of typical luminal (arrows) and basal cells (asterisks). All images in **B-D** with toluidine blue staining, scale bars 20 μm. (For interpretation of the references to colour in this figure legend, the reader is referred to the Web version of this article.)

The proximal, nodular secretory gland part of a Moll gland has a wide lumen (Figs. 5 and 6) with a layer of highly prismatic to cubic cells and a layer of basal cells surrounded by myoepithelial cells and is sitting on a basement membrane (Fig. 6). The height of the epithelial cells gives an indication of their state of activity. In particular, if cytoplasmic components are visible on the surface in the form of protrusions and are pinched off, the cells are maximally active (Fig. 6). If the cells are almost flat, this indicates an inactive state (Fig. 6). This pattern of activity has been described for other apocrine glands as well (Montagna, 1992). The terms active and inactive are usually based on morphological criteria (Schaffer, 1940), which can, however, be correlated with functional criteria (Welsch et al., 1998). The fine morphology of the Moll glands was intensively analyzed and documented by Stöckelhuber et al. in humans (Stoeckelhuber et al., 2003) (Table 2) and in monkeys (marmosets, rhesus monkeys and hamadryas baboon) (Stoeckelhuber et al., 2004) using histology, transmission electron microscopy and numerous protein markers by immunohistochemistry (Table 3 for the protein markers) as well as in pigs by Yasui et al. (2006). Some other authors have also detected proteins in the Moll glands (Table 3). However, it is not yet clear whether all these proteins (Table 3) detected in the active epithelial cells of the Moll glands are actually secreted and transported via the excretory duct.

Stöckelhuber et al. (Stoeckelhuber et al., 2003) as well as Jakobiec and Zakka (2011) were essentially able to confirm the morphological findings already made by Sattler (1877). In addition to the various stages of activity observed in the glandular epithelium, homogeneous pinched-off blebs were seen in the ducts, originating from the apex of the glandular cells. Stöckelhuber et al. (Stoeckelhuber et al., 2003), found no differences between the sexes and, in contrast to Ikeda (1953) (Table 1), observed no differences in the distribution of the Moll glands between the upper and lower eyelids or the various sections of the eyelids (temporal, middle, nasal) in humans. Unlike Old World monkeys, New World monkeys have significantly more Moll glands and the glands are located deeper in the eyelid (Stoeckelhuber et al., 2004).

**Fig. 7.**
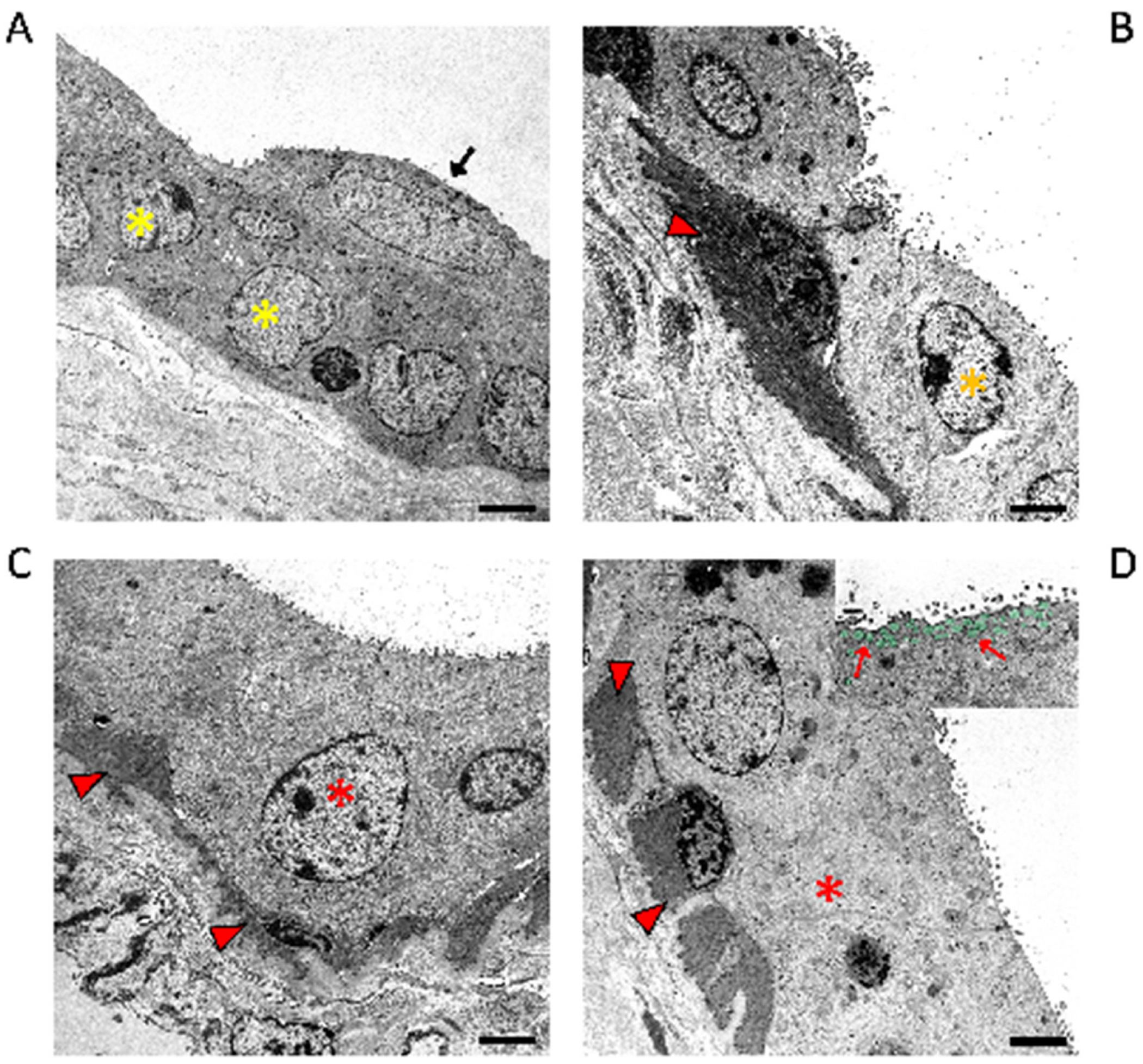
Transmission electron micrographs of a Moll gland with inactive and active glandular parts. **A** Depiction of the epithelium of an excretory duct consisting of two cell layers made up of typical luminal (black arrow) and basal cells (yellow stars on cell nuclei). **B** In the area of the glandular part, myoepithelial cells lie beneath the epithelium, which appear here as dark cells (red arrowhead). The myoepithelial cell appears with typical dense bodies and contractile filaments. The image was taken from the inactive area of a Moll gland with a single cell layer (orange-coloured star on cell nucleus). **C** A single layer of active secretory cells (red star on nucleus) and underlying myoepithelial cells (red arrowheads). The cytoplasm of the gland cells contains granules of different sizes with different electron densities and also luminescent lipid material. **D** Active apocrine gland cells (red star) of a Moll gland with high apical cells and secretory vesicles stained green in the inset and marked by red arrows. Bars 2,5 μm. (For interpretation of the references to colour in this figure legend, the reader is referred to the Web version of this article.)

Ultrastructurally, active Moll gland epithelial cells have tall apical protrusions with loosely distributed microvilli on the apical membrane (Stoeckelhuber et al., 2003) (Figs. 6 and 7). If apocrine protrusions are released according to the apocrine secretion mode, as already described by Sattler (1877), no microvilli are visible (Fig. 7). According to Stöckelhuber et al. (Stoeckelhuber et al., 2003), the apocrine protrusions can be up to 12 μm in size and, if they are very well developed, contain a few cell organelles and granules. Normally there are no organelles in the protrusions. The lateral cell membrane of the Moll gland epithelial cells is characterized by numerous interdigitating invaginations, which serve to enlarge the surface. The cells are connected to each other by tight and intermediate junctions, and there are also numerous desmosomes between the cells (Stoeckelhuber et al., 2003). The cytoplasm of the gland cells contains granules of different sizes with different electron densities and also luminescent lipid material, which could represent lipofuscin granules (Stoeckelhuber et al., 2003)(Fig. 7). The cytoplasm also contains abundant mitochondria and rough and smooth endoplasmic reticulum (Stoeckelhuber et al., 2003) indicating a high level of secretory activity in these cells.

The excretory ducts were already described in detail by Sattler (1877). Histologically and ultrastructurally, the excretory ducts of the Moll glands are lined by a two-layered cubic epithelium (Stoeckelhuber et al., 2003) (Figs. 6 and 7).

## 4. Cells involved in the structure of the Moll glands

Morphologically, Moll glands are formed by several different cell types (Jakobiec and Zakka, 2011; Stoeckelhuber et al., 2003; Yasui et al., 2006). The Moll glands consist of secretory cells, myoepithelial cells, and excretory duct cells, which work together to maximize the surface area within a limited volume, while a variety of stromal cells or connective tissues with extracellular matrix (ECM) support the Moll glands. The main components of the stromal connective tissue are not yet known, although it retains the embedded ductal network. From the apocrine active mammary gland, it is known that fibroblasts supporting the haematopoietic system, vascular endothelial cells supporting the blood vessels, a variety of innate immune cells (both macrophages and mast cells) and nerves are part of the gland function (Muschler and Streuli, 2010; Watson and Khaled, 2008). For sweat glands and their excretory ducts it is known that they originate from epidermal progenitors (Lu et al., 2012). However, all this is still completely unknown for the Moll glands.

## 5. Apocrine versus eccrine glands in mammals

Humans have around 2–4 million sweat glands distributed throughout the body (Baker, 2019). Their secretion is controlled by the central nervous system and the autonomic nervous system (Lu and Fuchs, 2014). The neurotransmitters acetylcholine and noradrenaline are mainly involved in their regulation. Depending on the sweat secretion, a distinction is made between 2 types of sweat glands: eccrine sweat glands (are distributed over the entire body; the excretory duct on the skin surface opens and releases the water- and salt-based secretion that is used for cooling) and so-called apocrine sweat glands (are located exclusively in the hairy areas of the body; are also called scent glands; the ingredients and their function are unclear) (Jakobiec and Zakka, 2011). The apocrine type of sweat glands is an appendage of the hair follicle with a thick, short duct connected to the upper hair follicles that secretes fluid into the hair canal before reaching the skin surface (Lu and Fuchs, 2014; Sato et al., 1989; Wilke et al., 2007). Apocrine sweat is a cloudy, viscous fluid containing proteins, lipids and steroids as well as water and electrolytes. It is initially odorless but can be processed by bacteria (Corynebaterium striatum) into smaller, odor-producing compounds (Preti and Leyden, 2010; Shehadeh and Kligman, 1963). Moll glands are attributed to the apocrine type of sweat glands but form a special type together with the ceruminous glands of the external auditory canal (Groscurth, 2002).

There are several indications that there are other types of sweat glands in addition to eccrine and apocrine glands. For example, apoeccrine or mixed sweat glands have been described in human axillary and perianal skin (Ito, 1988; Sato et al., 1987). They develop during puberty from eccrine-like precursor glands. The secretory spiral of the apocrine glands consists of two segments that are connected to each other: an expanded segment that is very similar to that of the apocrine glands and an unexpanded segment that cannot be distinguished from that of an eccrine secretory spiral (Groscurth, 2002). Another type of sweat gland with a unique morphology has been identified in the anogenital region of humans (van der Putte, 1993). It is characterized by a long excretory duct and a wide, tortuous excretory duct that tends to diverticulate and branch short and is lined by large, columnar lumen cells with conspicuous ‘trunks’. They differ clearly from the eccrine, apocrine and apoeccrine glands and are more similar to mammary glands (Groscurth, 2002). The function of the apocrine and anogenital sweat glands is still unclear.

Eccrine sweat glands make up 90 per cent of all sweat glands (~700/cm^2^ in adults on the palms and soles (Groscurth, 2002; Hwang and Baik, 1997; Szabo, 1967)). They are distributed over the entire body, except on the edges of the lips, nail beds, nipples, inner foreskin, labia minora, glans penis and glans clitoris. Their function is mainly thermoregulation, but they also play an important role in the response to emotional stimuli (Asahina et al., 2015). In contrast to the eccrine sweat glands, which are widely distributed over the body surface, apocrine sweat glands are limited to intensely hairy areas of the body such as the armpits, perianal region, areolas, periumbilical skin, foreskin, scrotum, mons pubis and labia minora (Groscurth, 2002; Sato, 1993). The ceruminal glands of the external auditory canal and the ciliary glands (Moll glands) of the eyelids are also usually included in this category but differ significantly with regard to several factors and should therefore be considered as separate subtypes (Groscurth, 2002). Apocrine sweat glands occur in much lower densities (~50/cm2 or less) than eccrine sweat glands (Lu and Fuchs, 2014; Sato et al., 1989). Beer et al. (2006) showed that all or most sweat glands are located in the subcutaneous tissue of the axilla of adult humans. While most domestic mammals lack eccrine sweat glands on the majority of their body surface, sweating remains crucial for thermoregulation in many species, helping them withstand extreme climatic conditions and stress. Camels are prime examples of working animals for whom sweating is vital for survival; they rely on apocrine gland secretion to dissipate heat (McEwan Jenkinson et al., 2006; Schmidt-Nielsen et al., 1957). The mouse, the most commonly used experimental animal, has eccrine sweat glands only in the pads of its feet and has no sweat glands on the skin of its trunk. Such animals are sensitive to extreme climatic conditions. Humans are the only mammals that have eccrine sweat glands over most of their body surface, while apocrine glands are found exclusively in heavily hairy regions, including the eyelid margin (Moll glands). The development of apocrine to eccrine glands is one of the most important characteristics that humans have acquired in order to gain a survival advantage against extreme climatic fluctuations.

## 6. Prenatal development of Moll glands

The rudiments of the apocrine sweat glands are formed at the same time as those of the eccrine glands (Groscurth, 2002; Lu and Fuchs, 2014). They develop as solid epithelial protrusions on one side of the sebaceous gland apparatus. Initially they are widely distributed over the body, but their number decreases from the fifth month of pregnancy (Groscurth, 2002). At birth, clearly defined glands can be found in some places, but they do not start secreting until puberty (Groscurth, 2002; Hashimoto, 1970; Hoepke, 1927; Sato et al., 1989). An exception to this is the Moll gland, which is already secretory active from birth (Sattler, 1877; Stoeckelhuber et al., 2003). Moll glands share this with the ceruminal glands in the external auditory canal, which are also active from birth (Requena et al., 1998), suggesting a modified function that must be active from birth. Using genome-wide analyses and functional studies in mice, it has been shown that sweat glands are differentiated by mesenchymal morphogenetic proteins (BMPs) and fibroblast growth factors, which signal to epithelial buds and suppress the production of sonic hedgehog (SHH), which is derived from the epithelium. Conversely, hair follicles are formed when mesenchymal BMP signaling is blocked, allowing SHH production. Fate determination in mice is restricted to a critical developmental window and is regionally specified (Lu et al., 2016). In contrast, in humans, a shift from hair to gland fates occurs when a BMP spike silences SHH during the last embryonic wave (s) of bud morphogenesis (Lu et al., 2016).

The further maturation of the apocrine sweat glands appears to be dependent on sex hormones, but not their maintenance, since gonadectomy in adults has no effect on their function (Groscurth, 2002). Stöckelhuber et al. (Stoeckelhuber et al., 2003) were able to detect androgen receptors in the human Moll glands they examined, while the estrogen receptor status was negative. However, it must be noted that the donor tissue was over 60 years of age, and therefore no statement can be made about the hormone receptor status before, during or shortly after puberty.

## 7. Function of the Moll glands

Stöckelhuber et al. (Stoeckelhuber et al., 2003) have speculated that the Moll glands could contribute to the tear film, but this is rather unlikely since they open into the hair funnels of the eyelashes (Takahashi et al., 2013) and the epithelial cells of the cornea and conjunctiva as well as the glands that contribute to the formation of the tear film also produce all of these defense proteins (Garreis et al., 2010; McDermott, 2009) which Stöckelhuber et al. (Stoeckelhuber et al., 2003) blames for this. Stöckelhuber et al. (Stoeckelhuber et al., 2003, 2004, 2008) have identified several defense proteins in the Moll glands in particular, such as IgA, lysozymes, lactoferrin, beta-defensins 1 and 2, and cathelicidin (LL37). Based on these findings, they hypothesize that one of the primary functions of the Moll glands could be to defend against bacteria and other pathogens present in the eyelid shaft and on the ocular surface. This hypothesis is further supported by the detection of hornerin in the Moll glands (Garreis et al., 2017), as hornerin is known to play a role in immune defense. The presence of these antimicrobial proteins suggests that the Moll glands may be actively involved in maintaining ocular surface homeostasis by providing localized immune protection against harmful microorganisms, particularly in areas exposed to external environmental threats. However, systematic studies of Moll gland secretions are lacking to support the hypothesis.

Because eyelashes form a barrier between the external and internal environments of the eye, they are extremely sensitive to a variety of threats and stimuli and are intensely innervated to perform this function (Aumond and Bitton, 2018; Montagna and Ford, 1969). The human lower eyelid contains 75 to 80 eyelashes in three to four rows, while the upper eyelid has 90 to 160 eyelashes in five to six rows. However, ethnic differences exist (Liotet et al., 1977; Montagna and Ford, 1969; Na et al., 2006; Singh et al., 2024). A good overview of the anatomy of eyelashes and hair and their similar features can be found in the review article by Aumond and Bitton (2018). The anatomy of eyelashes and hair shares some similar features (Aumond and Bitton, 2018; Marieb et al., 2005). Both have a hair shaft (the visible part) that extends outside the skin, a root that is located under the skin, and a bulb that is the enlarged terminal part. The lower part of the bulb is in direct contact with the dermal papilla, which enables important mesenchymal and epithelial interactions during the follicular cycle (Aumond and Bitton, 2018). The Moll glands open into the eyelash follicles, supplying them with their secretion. However, it is unclear whether each eyelash follicle has its “own” Moll gland (which would be assumed from an evolutionary perspective) or whether a single Moll gland can serve multiple follicles through several excretory ducts.

**Fig. 8.**
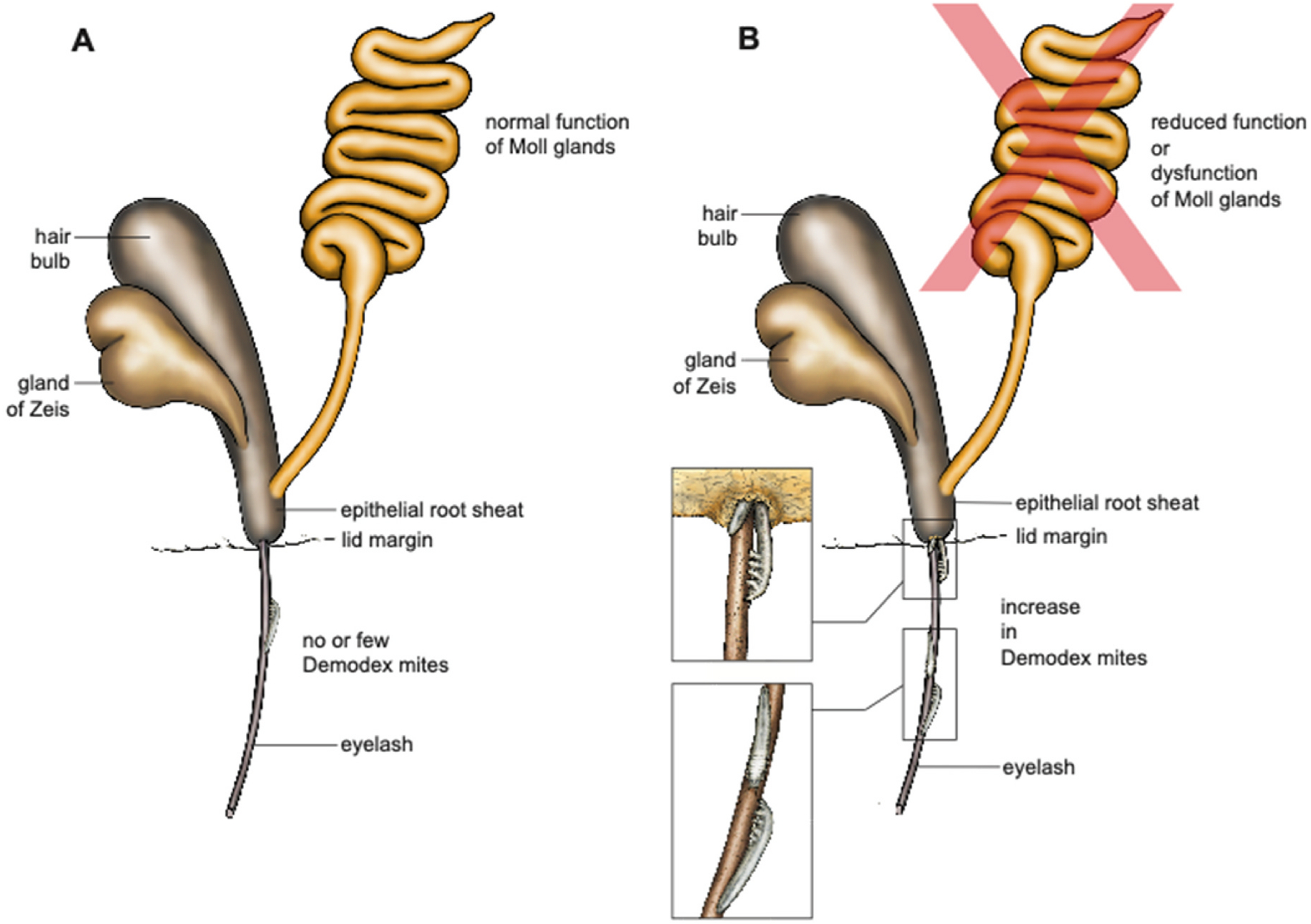
Schematic representation of an eyelash with Zeis gland and Moll gland opening into the hair funnel. **A** If the Moll glands function normally, the secretion products of the Moll glands could hypothetically contribute to the fact that no or only very few Demodex mites appear on the eyelashes and support the homeostasis of the eyelids. This theory needs to be scientifically tested in the future (drawing by Jörg Pekarsky). **B** Reduced Moll gland function (red X on the Moll gland) or dysfunction of the Moll glands thus hypothetically could contribute to a reduced defence against Demodex mites, which multiply and contribute to inflammatory reactions at the eyelid margin (blepharitis) (drawings by Jörg Pekarsky). (For interpretation of the references to colour in this figure legend, the reader is referred to the Web version of this article.)

The eyelashes are a crucial component of the eyelid margin anatomy, along with the Meibomian glands, the eyelid skin and the biofilm, all of which contribute to the overall homeostasis of the ocular surface (Aumond and Bitton, 2018). Maintaining the integrity of these structures is therefore essential. The eyelid margin plays a key role in distributing the lipid layer of the tear film and protecting the eye from external influences. Inflammation of part of the eyelid margin, as seen in conditions like blepharitis (Yeu et al., 2024), can lead to disruption or instability of the tear film, negatively impacting the ocular surface (Craig et al., 2017). If left untreated, this inflammatory cascade can develop into dry eye disease (Aumond and Bitton, 2018; Craig et al., 2017). Recent reports show that 60–70 % of patients with dry eye disease also have Demodex blepharitis (Cheng et al., 2021; Trattler et al., 2022). Demodex mites, the most common ectoparasites of humans (25 million people are affected in the United States alone (Rhee et al., 2023)), are associated with blepharitis. Two species, Demodex folliculorum and Demodex brevis, are found on human skin, particularly on the eyelids. Both species are translucent, elongated, microscopic mites with four pairs of short, claw-like legs (Fig. 8). Demodex folliculorum is about 0.3–0.4 mm long and is found in clusters around the eyelash root and lash follicle, where it feeds on sebum and follicular epithelial cells (Fromstein et al., 2018). Demodex brevis is shorter, solitary, and prefers sebaceous glands (Fromstein et al., 2018). Although Demodex mites can be found on healthy, asymptomatic people of all ages (Biernat et al., 2018)(Fig. 8), it is suspected that they may play a pathogenic role when the mites reach higher densities and reach a state of demodicosis (Fromstein et al., 2018). Demodex blepharitis is equally common in both sexes and is similar regardless of ethnicity (Biernat et al., 2018; Rhee et al., 2023; Trattler et al., 2022). The prevalence of Demodex increases with age, affecting more than 80 % of those over 60 years of age and 100 % of those over 70 years of age (Cheng et al., 2015). On this basis, we hypothesize that the secretions of the Moll glands may play an important role in the defense against Demodex and thus contribute to the absence or low numbers of Demodex mites on the eyelashes, which support eyelid homeostasis, as shown in Fig. 8A, a function that declines with age and contributes to Demodex blepharitis with increasing age (Fig. 8B). Although plausible, these theories have yet to be scientifically tested.

In addition to their role in self-protection and immune defense, the secretion of the Moll glands can also be crucial for maintaining the homeostasis of the eyelid margin. This secretion likely contributes to the proper functioning of the eyelid, particularly in facilitating smooth and healthy eyelid movement during eyelid closure. The presence of these secretions may also help lubricate the eyelid margin, reducing friction and wear during the blinking process. Thus, the Moll glands could also play a role in maintaining the integrity of the tear film and protecting the ocular surface from potential damage. But even this is just a theory that has yet to be scientifically verified.

## 8. Thermoregulation

**Fig. 9.**
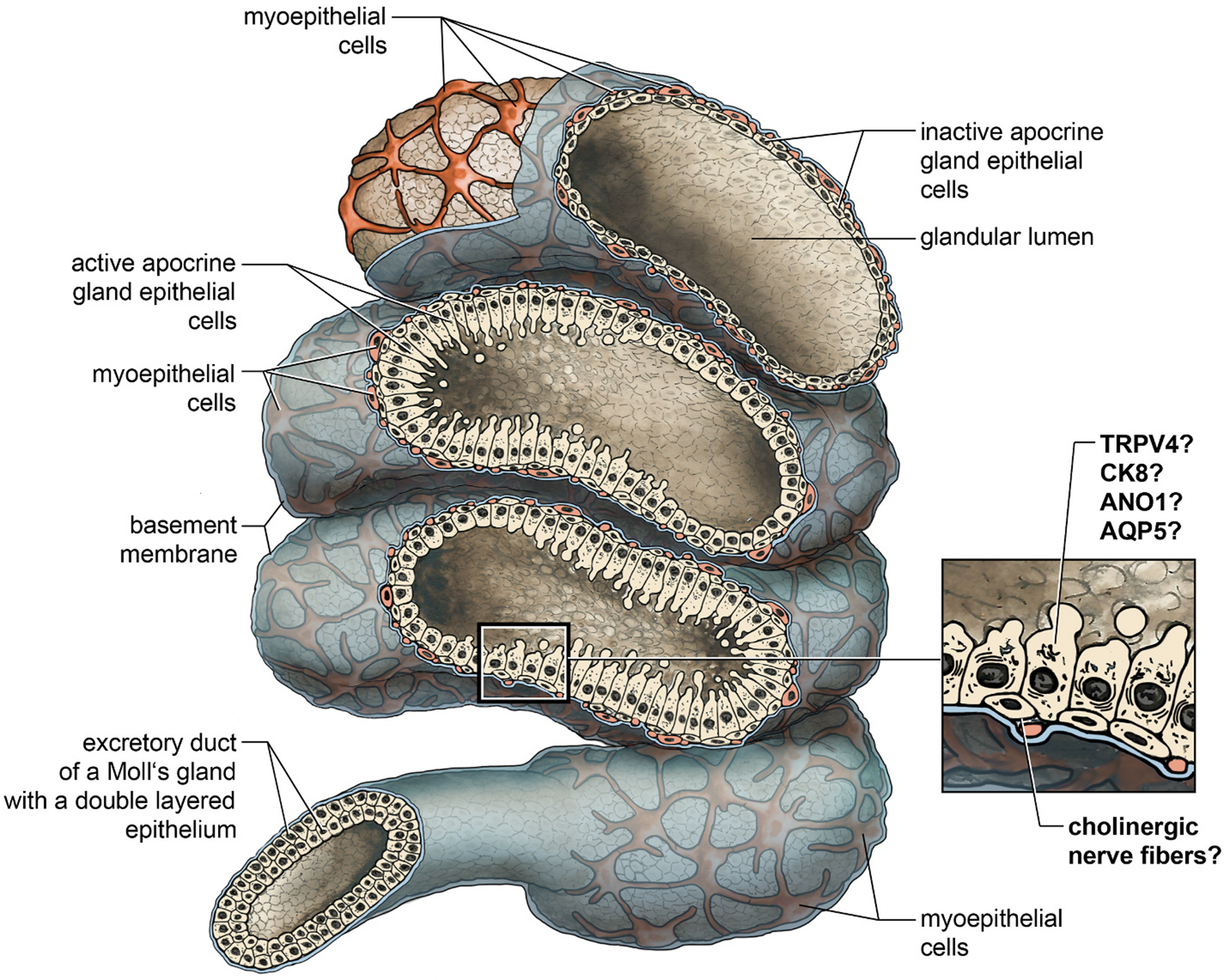
To date, it is completely unknown whether Moll glands have a thermoregulatory function at all and if so whether central proteins that are important for thermoregulation in sweat glands such as the transient receptor potential cation channel subfamily V member 4 (TRPV4) channel, the voltage-gated calcium-activated anion channel anoctamine 1, the water channel aquaporins 5 and others have a thermoregulatory function for Moll glands and which nerve fibres stimulate the secretion of Moll glands. The schematic illustration shows a Moll gland in high magnification with a section magnification in which this hypothesis is shown (drawing by Jörg Pekarsky). Further research must clarify whether Moll glands are involved in maintaining the health at the lid margin and maybe also the ocular surface.

In order to be able to adapt intensively to climatic conditions, humans have evolved to develop eccrine sweat glands and a strong reduction in apocrine sweat glands (Lu and Fuchs, 2014). As already mentioned above, some mammals such as camels or horses, whose sweating function is crucial for their survival and performance, only use apocrine secretions to dissipate heat (Lu and Fuchs, 2014; McEwan Jenkinson et al., 2006; Schmidt-Nielsen et al., 1957). Although various eccrine sweat glands are also found on the eyelid, it is hypothetically conceivable that the Moll glands also play an important role in thermoregulation, working together with the eyelashes, the eyelid margin and blinkrate to regulate extreme temperatures (Nagymihályi et al., 2004). Thermoregulation of the eyelid has a significant impact on the release of Meibomian gland secretion. For example, the secretion of the Meibomian glands is only liquid in a certain temperature range (Nagymihályi et al., 2004; Rosenfeld et al., 2013; Terada et al., 2004); it is possible that a similar thermoregulatory mechanism applies to the Zeis glands, which are located adjacent to the Moll glands, although there is currently no available data to confirm this. If the Moll glands are thermoregulatory, their mechanism of action would likely differ from that of the eccrine sweat glands, which are well-known for their involvement in temperature regulation through sweat production. Here, reports indicate that an interaction between TRPV4 and anoctamin 1 (ANO1) may be extensively involved in water efflux from exocrine glands, suggesting that this interaction may play a role in transpiration (Kashio et al., 2024). Therefore, it will be interesting to test the hypothesis and to study central proteins that are important for thermoregulation, such as TRP channels, aquaporins or ANO1, in detail in Moll glands (Fig. 9). Whether the Moll glands have a thermoregulatory function at all and if so how this works including their innervation pattern must be the subject of future research and could provide valuable insights into the complex processes involved in maintaining the health at the lid margin and maybe also the ocular surface. Another hypothesis to be investigated would be whether the secretions of the Moll glands contribute to altering the secretion of the Zeis glands so that it is better distributed on the lid margin and eyelashes or whether it is the mixture of Moll and Zeis glands secretions that has a particular effect (perhaps it would therefore be better to speak of an eyelash Moll-Zeis unit?). All of this needs to be investigated in the future and will not be easy to investigate, as there is not only uncertainty about the Moll glands but also about the secretion products and the functional significance of apocrine sweat glands for human skin as a whole (Lüllmann-Rauch and Asan, 2024). In this context, it is certainly interesting to note - as already mentioned above - that the Moll glands together with the ceruminous glands in the area of the external auditory canal are already active in childhood, in contrast to the apocrine scent glands, and are not only activated during puberty.

## 9. Communication of psychological state, emotional sweating

The sweat of the apocrine glands contains, among other things, pheromones and odor signals (Beer et al., 2006; Beier et al., 2005). Chemically speaking, pheromones are volatile steroid molecules (Cowley and Brooksbank, 1991; Grosser et al., 2000; Sobel et al., 1999). The secretory activity of the apocrine glands (with the exception of the Moll glands and the apocrine glands in the external auditory canal, see above) only begins in puberty (Groscurth, 2002; Hoepke, 1927). They have several functions in the human body. For example, they activate certain regions of the brain, particularly the limbic system (Sobel et al., 1999) and influence social behavior (Cowley and Brooksbank, 1991; Cutler et al., 1998; Grosser et al., 2000). They also regulate ovulation (Stern and McClintock, 1998), as well as physiological parameters such as serum levels of testosterone, luteinizing hormone (LH) and follicle stimulating hormone (FSH) and, in a sex-specific manner, respiration and heart rate (Monti-Bloch et al., 1998; Shinohara et al., 2000).

Other authors take a more critical view of the existence of pheromones in humans (Precone et al., 2020). Communication via pheromones is important in invertebrates and non-human vertebrates and also seems to play a role in humans (Gelstein et al., 2011). However, most genes that code for pheromone receptors are inactive in humans (Precone et al., 2020). It remains uncertain whether humans possess functional pheromone receptors. Pheromones are typically detected via vomeronasal receptors, but in humans, these receptors regress almost completely after birth (Shirokova et al., 2008). However, the genes that are switched on probably have a biological function (Precone et al., 2020). Additionally, pheromones have the potential to activate brain areas such as the hypothalamus (Precone et al., 2020). Whether Moll glands are involved in any way in the formation of such substances is still completely unclear.

However, only humans can shed emotional tears (Gelstein et al., 2011; Messmer, 2009; Murube et al., 1999; Murube, 2009a, 2009b). Their production seems to involve the same nerves, receptors and transmitters as basal and reflex tears (Messmer, 2009). However, the stimuli must be received in a cognitive/social context, recognized by “induction centers” in the telencephalon and transmitted to effector centers. Increased concentrations of proteins such as prolactin, manganese, potassium and serotonin have been detected in emotional tears, none of which have yet been studied in Moll glands. Various theories attempt to explain the cause and benefit of tears (Gelstein et al., 2011). A number of factors such as ethnicity, social status, occupation, hormonal situation, gender and individual threshold influence whether someone “cries” or not (Messmer, 2009). Further studies are needed to clarify whether the Moll glands’ role in this context is important. In the external auditory canal, the secretions of the ceruminal glands mix with the sebaceous gland secretions and eccrine sweat gland secretions, producing a bitter-tasting secretion that prevents microbial invasion (Stoeckelhuber et al., 2006) as well as insects and beetles from entering. The secretion of the Moll glands released onto the eyelashes could also have a similar function at the edge of the eyelid.

In addition, it should be discussed whether some of the proteins produced by the Moll glands may turn out to be interesting biomarkers that may also play a role in ocular surface diseases. Tear fluid has become increasingly important in recent years due to advances in detection methods for measuring ultra-low biomaker levels from low sample volumes (Gijs et al., 2025). In this context, an international task force called Tear Research Network was formed in 2022 (www.tearrese archnetwork.com) with the aim of advancing the field of tear fluid research. It is currently unclear whether the secretion of the Moll glands or parts of it enter the tear film. However, it is quite possible. A comparison of the Moll gland secretion with other gland secretions (e.g. lacrimal gland, salivary glands and others) and subsequent comparison with tear fluid could provide interesting new aspects as to whether the Moll glands produce certain proteins that are only produced by them or proteins that change significantly in the context of ocular surface changes such as dry eye disease, although the collection of fluid samples from Moll glands will likely be a challenge. The secretory nature of Moll glands in the form of small vesicles makes them an interesting candidate for analyzing extracellular vesicles (EVs), which are currently the subject of intensive research (Amorim et al., 2022; Chatterjee and Singh, 2023; Cross et al., 2023; Gijs et al., 2025; Hefley et al., 2022; Martins et al., 2024; Shiju and Yuan, 2024; van der Merwe and Steketee, 2017; Welsh et al., 2024; Yeung et al., 2022). Several EV cargo molecules have already been identified in tear fluid that act as potential biomarkers for dry eye disease, Sjögren’s syndrome and other eye-associated diseases (Shiju and Yuan, 2024). To date, there are no findings in this context for the Moll glands.

## 10. Nervous control and secretion mechanism

Apocrine gland cells are activated by sexual arousal and emotional stress (Hu et al., 2018). During sleep, these glands are normally inactive (Slominski et al., 2012). Whether this also applies to the Moll glands is not yet clear and still needs to be investigated. Regarding the surface of the eye, it is at least known that there are large differences in tear film proteins between the open eye and the closed eye (during sleep) (Sack et al., 2005, 2007), which indicates differences in the activity of various cells on the surface of the eye.

Apocrine sweat glands are innervated by sympathetic nerve fibers originating from the same spinal cord segments as the eccrine glands (Sato, 1993). However, unlike eccrine glands, which primarily use acetylcholine as a neurotransmitter, apocrine glands rely on catecholamines, with cells being more responsive to adrenalin (epinephrine) than to noradrenaline. Most unmyelinated nerve fibers terminate near the capillaries, rather than directly interacting with the secretory or myoepithelial cells, suggesting that apocrine sweat glands are regulated by a neurohumoral mechanism rather than direct neuronal stimulation (Groscurth, 2002). Harker proposes a dual activation of the apocrine sweat glands (Harker, 2013). In general, β-adrenergic stimulation is more effective than α-adrenergic stimulation (Robertshaw, 1974). Furthermore, the secretory part of the apocrine glands contains β2 and β3 adrenoreceptors near the basolateral membrane (Lindsay et al., 2008). Thus, these adrenergic receptors may mediate apocrine sweating in response to cholinergic stimuli. The myoepithelial cells, which also carry adrenergic receptors, could then squeeze the existing sweat out of the glands (Harker, 2013; Ishikawa et al., 1998). Unfortunately, there is limited information available on the detailed innervation of the Moll glands in this context with the exception of a study by Seifert and Spitznas (1999), who were able to detect vasoactive intestinal polypeptide (VIP) around Moll glands. VIP is a neuropeptide that functions both as a neuromodulator and neurotransmitter, playing a crucial role in regulating various physiological processes. It is a potent vasodilator that regulates smooth muscle activity, epithelial cell secretion and blood flow for example in the human minor salivary glands (Sato et al., 2022). Further research is needed to better and deeper understand the innervation and potential neurochemical influences on the Moll glands.

## 11. Homeostasis and wound healing

It has long been suggested that the sweat duct may serve as a “matrix for replacement” of glandular cells (Lobnitz et al., 1954). Several studies using lineage tracing and mouse models have shown that stem cells from both the epidermis and hair follicles contribute to the repair of skin wounds (Ito et al., 2005; Levy et al., 2007; Nowak et al., 2008; Snippert et al., 2010). Since there are more sweat glands than hair follicles in human skin, it has been hypothesized that another source of progenitor or stem cells within the sweat apparatus (ducts and glands) must contribute to the repair of skin wounds (Biedermann et al., 2010; Brouard and Barrandon, 2003; Lu and Fuchs, 2014).

There is a particularly large amount of knowledge about the homeostasis and wound healing of eccrine sweat glands from the mouse (Lu and Fuchs, 2014). Cellular self-renewal takes place in the glandular basal cells and the basal cells of the sweat gland ducts. In addition, obsolete suprabasal epidermal cells and intraepidermal duct cells are also replenished from here (Lu and Fuchs, 2014). When epidermal injury and loss occurs near a gland, there is an increase in proliferation that occurs in the neighboring healthy basal cells of the sweat duct and epidermis. These cells, along with their descendants, rapidly migrate and differentiate to repair the damaged area. While the progenitor cells in the glands are primarily unipotent, it is thought that there are also a small number of multipotent stem cells that come into play when extensive remodelling or regeneration is required (Lin and Lu, 2021). The active secretory epithelial cells of the sweat glands, on the other hand, do not respond to injuries to the epidermis (Lu and Fuchs, 2014). If cells in the sweat gland are injured, the neighboring healthy cells can be activated to repair locally. If luminal cells are injured during a gland injury, the neighboring luminal cells proliferate to repair (Lu and Fuchs, 2014). Injured myoepithelial cells are replaced by neighboring myoepithelial cells (Lu and Fuchs, 2014). Recent studies in mice show a mutual interaction between the vascular niche and the sweat gland for this purpose (Yuan et al., 2023). Similar mechanisms can be assumed for apocrine sweat glands in general and for Moll glands in particular, but there are no findings to date, although they would be helpful for processes that take place in the area of the eyelid margin. It should also be critically noted that most of the studies originate from mice, which, according to Ikeda (1953) (Table 1), have no Moll glands at all. Nevertheless, due to the large number of Moll glands in humans, more should be found out about their function and regulation with regard to homeostasis and wound healing mechanisms.

## 12. Pathological states

A deeper understanding of the Moll gland function is also desirable from a clinical point of view. It is well known that bacterial inflammation of a Moll gland, as well as bacterial inflammation of a Zeis or Meibomian gland, leads to hordeulum externum (Sun et al., 2019). The risk of hordeolum is increased in patients with a history of Meibomian gland dysfunction (MGD) and blepharitis. Moll glands can, although very rarely, develop into neoplasms (Barker-Griffith et al., 2006). Benign tubular apocrine adenomas of the eyelid have been described (Eiger-Moscovich et al., 2019; Jakobiec and Zakka, 2011). In addition, Moll glands can be the site of origin of apocrine adenocarcinomas (Dusseldorp, 1929; Gates et al., 1943; Hagedoorn, 1937; Seregard, 1993; Stout and Cooley, 1951; Whorton and Patterson, 1955; Zeeman, 1923). This is also a very rare neoplasm of the gland cells that mainly affects older people, with a predominance of men (Rosales Santillan et al., 2019). Clinically, it appears similar to a stye in the eye, with which it is often confused (Rosales Santillan et al., 2019). Whether disorders of Moll gland function may also be a cause of eyelash loss or eyelash changes is completely unknown and needs to be investigated in the future experimentally.

## 13. Conclusions and future perspectives

This review makes it clear that our knowledge of the Moll glands, which occupy a considerable volume in the eyelids, is extremely limited. Despite their obvious importance, there is basically only descriptive information on their morphology and some cellular contents/secretion products. The rest is speculation and no reliable data regarding their functions. Further research, especially basic science studies, is essential to gain a deeper understanding. In addition, clinical morphologic studies are crucial to investigate whether changes in the Moll glands are associated with common eye diseases such as dry eye disease and meibomian gland dysfunction. These diseases are known to disrupt the tear film and affect the overall health of the eye. Understanding the effects on the Moll glands and vice versa could provide valuable insight into the contribution of the glands to these pathologies and could also provide indications of good biomarkers. Deeper insights would also potentially help to better understand changes and diseases of eyelashes and the lid margin and help identify potential therapeutic targets to improve ocular surface health. This would also be highly desirable in view of the large amount of lifestyle products used on the eyelid margin and eye (Craig et al., 2023) which we have not discussed further here.

Possible questions that could advance Moll gland research would be, for example, to clarify which animals would be useful for experimental studies. Apart from those studied by Ikeda (1953) (Table 1), pigs have Moll glands and are suitable for experiments. Pigs would offer a possibility for *ex vivo* studies, as they would be readily available from slaughterhouses. Conventionally used laboratory animals such as mice, rats or rabbits are ruled out due to Ikeda (1953) because they do not have Moll glands. Which genes and factors are important for the development of Moll glands? What do Moll glands look like in mild and severe cases of dry eye disease? This could be investigated in dogs who also can develop dry eye disease (Hisey et al., 2023). Analysis of the secretome of Moll glands? Examination of old body donors in relation to tissue samples from younger individuals, whether there is a change in Moll gland morphology with increasing age, etc. There are so many questions to be answered in this context that it will not be difficult to engage in intensive scientific research. This review is intended to encourage this.

In summary, we can say that our review article has shed some light on the person who gave the Moll glands their name, that we however know much to less about the Moll glands themselves and that great efforts are needed to be made to close this knowledge gap as quickly as possible.

## Supplementary Material

Supplemental figures 1-3

## Figures and Tables

**Table 1:** Occurrence and distribution of Moll glands in different species (Ikeda, 1953). X = glands are present, 0 = glands are not present. For future studies on the Moll glands, it is noteworthy that common laboratory animals such as mice, rats and rabbits do not appear to have Moll glands, whereas they are found in slightly larger species.

spezies	occurrence	strongest occurrence
human	X	Middle part of upper eyelid
monkey	X	Medial and middle part of upper eyelid
cattle	X	Middle part of upper eyelid
horse	X	Middle part of upper eyelid
pig	X	Middle part of upper eyelid
dog	X	Medial part upper eyelid
cat	X	Medial part upper eyelid
rabbit	0	
guinea pig	0	
rat	0	
mouse	0	
hamster	0	

**Table 2:** Staining characteristics in the secretory cells of human Moll’s glands done so far with conventional dyes and lectins (Stoeckelhuber et al., 2003).

Dye	Staining

Nile blue	Supranuclear granules stained in active and inactive epithelial gland cells.
PAS reaction	Weak or negative staining in cells, strong reaction in granules and luminal material.
Alcian blue (pH 2.5)	Strong apical staining in inactive cells and active cell protrusions.
**Lectin**	**Staining**
Canavalia ensiformis agglutinin (ConA)	Weak to medium cytoplasmic staining, strong apical staining.
Helix pomatia agglutinin (HPA)	Medium intensity throughout the cell, strong apical staining.
Ricinus communis agglutinin I (RCAI)	Weak to no cytoplasmic staining, strong apical staining and luminal material.
Wheat germ agglutinin (WGA)	Strong apical staining in both active and inactive cells.
Peanut agglutinin (PNA)	Variable cytoplasmic staining, strong apical reaction.
Ulex europaeus agglutinin I (UEAI)	Intensive apical staining in some cells, weak cytoplasmic staining.

**Table 3:** Proteins investigated in the secretory cells of human Moll glands. The table summarizes what is currently known about the secretion products build by Moll glands. However, whether these are actually secreted is unknown and needs to be analyzed further.

Abbreviation	Protein name	Description	Distribution in Moll’s glands	References
IgA	Immunoglobulin A	role in the immune function of mucous membranes	Variable reactivity: Granular IgA in some cells, apical staining in others.	Stoeckelhuber et al. (2003)
SC of IgA	Secretory component of immunoglobulin A	a proteolytic cleavage product of the polymeric immunoglobulin receptor which remains associated with dimeric IgA in sero-mucous secretions	Strong apical reactivity and IgA-positive secretory material in the lumen.	Stoeckelhuber et al. (2003)
lysozyme	Lysozyme	a small (14-kDa) cationic protein that exerts antibacterial activity through its ability to hydrolyze peptidoglycans in the bacterial cell wall	Variable reactivity, often in granules and positive in luminal material.	Stoeckelhuber et al. (2003)
MUC1	Mucin 1	role in mucus, and involvement in cell signaling	Weak to medium cytoplasmic reactivity, strong apical reactivity.	Stoeckelhuber et al. (2003)
AR	Androgen receptor	a type of nuclear receptor activated by androgenic hormones, including testosterone and dihydrotestosterone (DHT) in the cytoplasm and then translocating into the nucleus	Only a few cells show positive nuclear reactivity.	Stoeckelhuber et al. (2003)
ER	Estrogen receptor	mediates the effects of estrogenic compounds on its target tissue	*No nuclear reactivity*.	Stoeckelhuber et al. (2003)
actin	Actin	protein that is an important contributor to the contractile property of muscle and other cells	Medium to strong reactivity in all secretory cells, stronger apically in active cells.	Stoeckelhuber et al. (2003)
CK19/CK7	Cytokeratin 19/7	is a 40 kDa protein that in humans is encoded by the FRT19 gene	Weak to strong cytoplasmic reactivity, stronger at the base of apical protrusions.	Stoeckelhuber et al. (2003)
hBD-1	Human beta-defensin 1	skin inflammation and/or skin responsiveness to any kind of allergic reactio	Weak to moderate cytoplasmic reactivity, strong apical reactivity, especially in protrusions; lumen material strongly reactive; some granules react intensely in active cells.	Stoeckelhuber et al. (2004)
hBD-2	Human beta-defensin 2	broad-spectrum activity against gram-positive and gram-negative bacteria and kill bacteria in a number of ways	Moderate reactivity in the entire epithelium, slightly more intense in protrusions; positive luminal reactivity; fine granular pattern observed at higher magnification in active cells.	Stoeckelhuber et al. (2004)
LL37	Cathelicidin	belongs to a third general class of epithelial antimicrobial peptides that are expressed in epithelia and kill microorganisms by membrane disruption	Moderate reactivity in the cytoplasm and protrusions; luminal material positive; some granules show intense apical reactivity in active cells.	Stoeckelhuber et al. (2004)
GCDFP-15 (PIP, SABP)	Gross cystic disease fliud protein-15	Prolactin-induced protein normally is found in the saliva and other bodily secretions of mammals, but can also be detected in pathological changes in the mammary glands	Strong reactivity in the apocrine secretory spiral of Moll glands, no reactivity in the apocrine duct.	Jakobiec and Zakka (2011)
α-SMA	Alpha-smooth muscle actin	is a smooth muscle protein that is responsible for cell structure, motility and contractility	Strong reactivity in the apocrine secretory spiral of Moll glands, no reactivity in the apocrine duct.	Jakobiec and Zakka (2011)
CK7 (KRT7)	Cytokeratin 7	cytokeratin or brief keratin 7, is a type II keratin that is found in various epithelial tissues of the human body.	Strong reactivity in the apocrine secretory spiral of Moll glands, no reactivity in the apocrine duct.	Jakobiec and Zakka (2011)
EMA	Epithelial membrane antigen	immunohistological marker that is expressed by many epithelial cells; belongs to the cytokeratins;in its soluble form, which occurs in blood plasma, it is known as cancer antigen 15-3 (CA15-3)	Strong reactivity in the apocrine secretory spiral of Moll glands, some reactivity in the apocrine duct.	Jakobiec and Zakka (2011)
CEA	Carcinoembyonic antigen	protein that is produced in the intestine, liver, pancreas, mammary gland and others, and can be measured in the blood; important tumor marker	Strong reactivity in the apocrine secretory spiral of Moll glands, some reactivity in the apocrine duct.	Jakobiec and Zakka (2011)
HRNR	S100 fused-type protein hornerin	S100 fused-type protein that plays a role in the epidermal differentiation complex that is involved in terminal differentiation of keratinocytes	Cytoplasmic reactivity of epithelial cells.	Garreis et al. (2017)
FLG2	S100 fused-type protein filaggrin-2	essential for normal cell-cell adhesion in the cornified cell layers	*No reactivity*.	Garreis et al. (2017)
SFTA3/SP-H	Surfactant protein H	surfactant protein with physicochemical properties	Cytoplasmic reactivity of epithelial cells.	Schicht et al. (2018)
UT-A	Urea transporter A	membrane transport protein, transporting urea	Marked UT-A signals in the tubular epithelium, especially in the apical cell compartments.	Jäger et al. (2022a)
UT-B	Urea transporter B	membrane transport protein, transporting urea	Pronounced UT-B-specific immunoreactivity in the tubular epithelium, especially in the apical parts of the cells.	Jäger et al. (2022b)

## Data Availability

Data will be made available on request.

## Footnotes

1 Franciscus (Frans) Cornelius Donders (27.05.1818–24.03.1889) was a Dutch physiologist, anatomist and ophthalmologist. He held a professorship in physiology in Utrecht and was internationally recognized as an authority on eye diseases. He headed the Netherlands Hospital for Eye Patients. Together with Albrecht von Graefe (22.05.1828–20.07.1870/Berlin/Germany) and Hermann von Helmholtz (31.08.1821–08.09.1894/Berlin/Germany), he was one of the founders of science-based ophthalmology and, together with his friend Albrecht von Graefe, founded the first scientific eye journal ‘Archiv für Augenheilkunde’, which Donders and Ferdinand von Arlt (18.04.1812–07.03.1887/Prague/Czech and Vienna/Austria) renamed Graefe’s Archiv für Augenheilkunde after the death of Graefe (today Graefe’s Archiv for Clinical and Experimental Ophthalmology).

## Appendix A. Supplementary data

Supplementary data to this article can be found online at https://doi.org/10.1016/j.preteyeres.2025.101362.

## Data availability statement

All data presented in this study are available on request from the corresponding author (F.P.).
