# Corrigendum to “Glands of Moll: history, current knowledge and their role in ocular surface homeostasis and disease” [Progr. Retin. Eye Res. 106 (2025) 101362]

**DOI:** 10.1016/j.preteyeres.2025.101364

**Published:** 2025-05-21

**Authors:** Michael Stopfer, Ingrid Zahn, Katharina Jüngert, Gerhard Aumüller, Frans L. Moll, Martin Schicht, Helen P. Makarenkova, Cintia S. de Paiva, Friedrich P. Paulsen

**Affiliations:** a Institute of Functional and Clinical Anatomy, Friedrich-Alexander-University Erlangen-Nürnberg, Universitatsstr. 19, Erlangen, Germany; b Philipps-University Marburg, Am Möhrengarten 1, Münchhausen, 35117, Germany; c Department of Vascular Surgery, University Medical Center Utrecht, Heidelberglaan 100, 3584 CX, Utrecht, the Netherlands; d Department of Molecular Medicine, The Scripps Research Institute, 10550 N Torrey Pines Rd, La Jolla, CA, 92037, United States; e Ocular Surface Center, Department of Ophthalmology, Baylor College of Medicine, One Baylor Plaza, Houston, TX, 77030, United States

The authors regret that.

1. There is a **mistake in** Fig. 2. Please change this figure with the attached figure.

**Figure:**
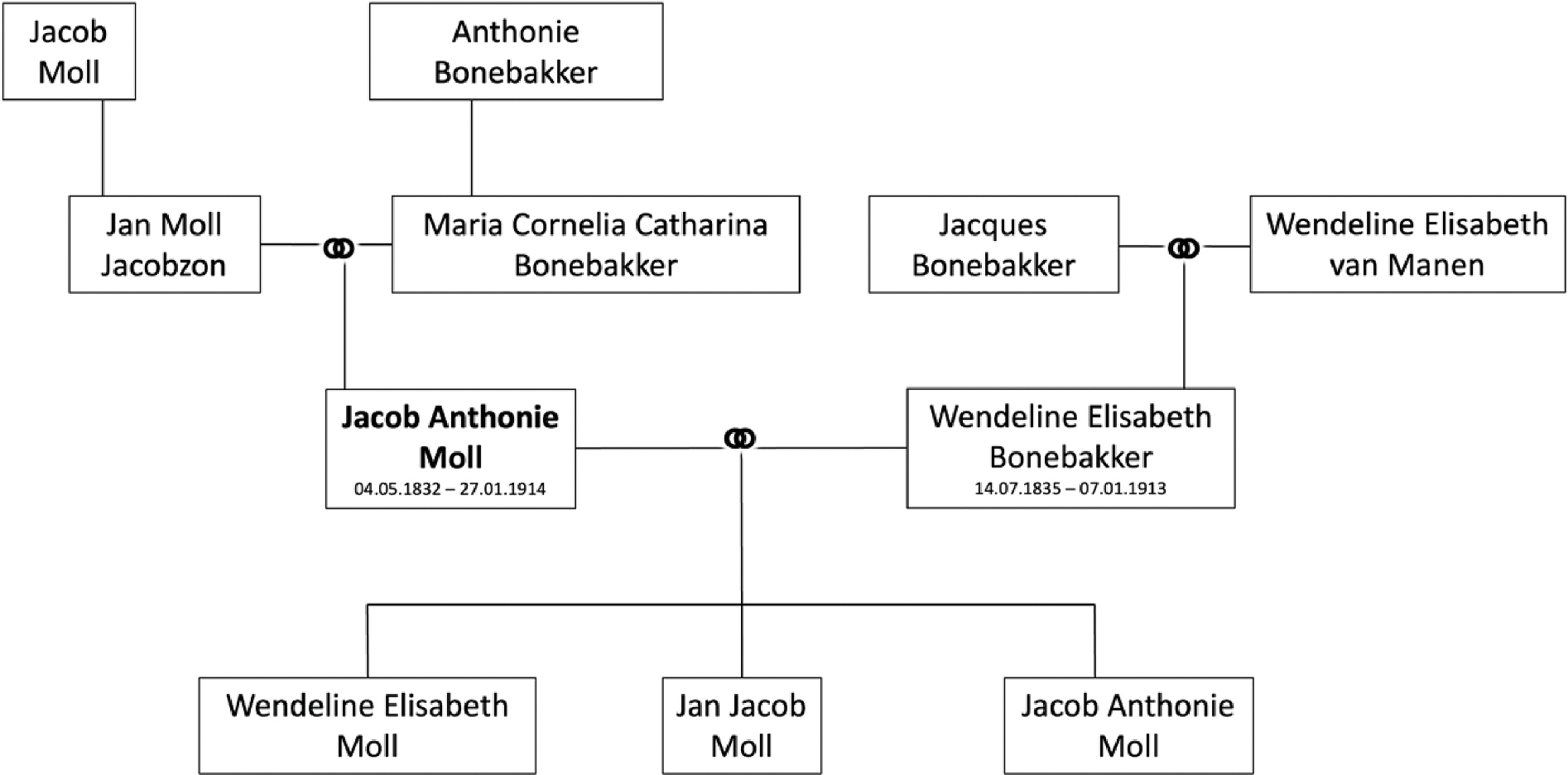


3. We forgot to mention under “Acknowledgments”: The present article is part of the first author’s (M.S.) doctoral thesis. The underlying work was performed in fulfilment of the requirement for obtaining the degree at the Medical Faculty of the Friedrich-Alexander-Universität Erlangen-Nürnberg.

The authors would like to apologise for any inconvenience caused.

