# Supplementary figures and images for: Glands of Moll: history, current knowledge and their role in ocular surface homeostasis and disease
Source: Prog Retin Eye Res. Author manuscript; Available in PMC 2025 Sep 30. (PMC12483061; doi:10.1016/j.preteyeres.2025.101362)

**Suppl. Fig. 1**

**
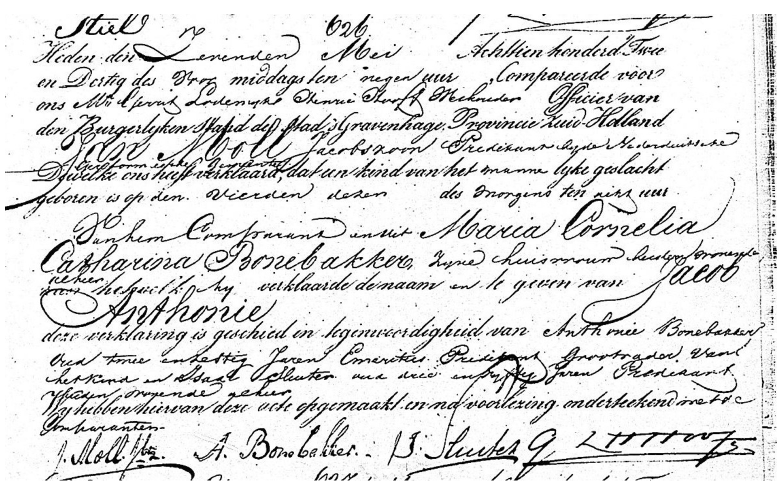
**

**Suppl. Fig. 2**

**
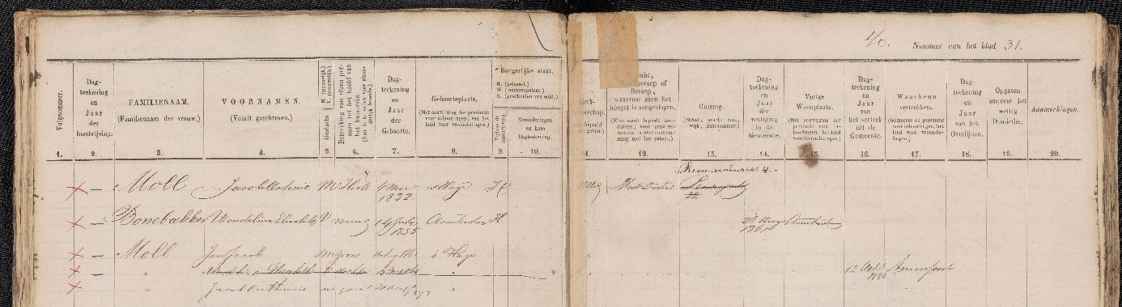
**

**Suppl. Fig. 3**

**
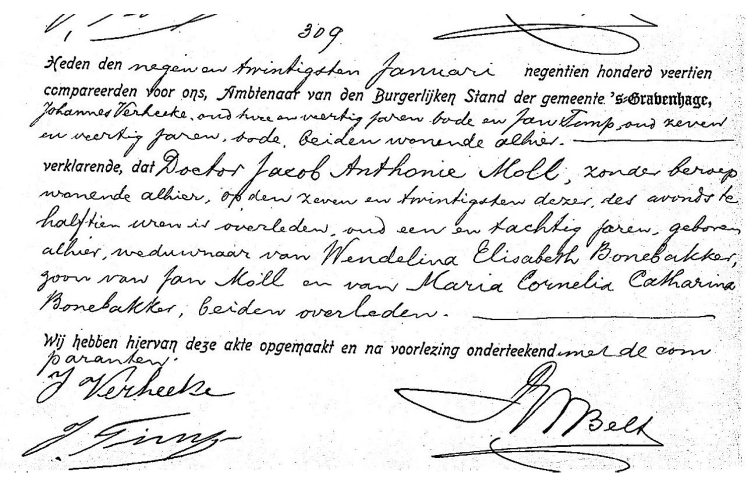
**

Supplement: Supplemental figures 1-3 [file NIHMS2112771-supplement-Supplemental_figures_1-3.docx]
